# Catalyst characterization in the presence of solvent: development of liquid phase structure–activity relationships

**DOI:** 10.1039/c7sc03728g

**Published:** 2017-11-16

**Authors:** Nicholas S. Gould, Bingjun Xu

**Affiliations:** a Catalysis Center for Energy Innovation , Department of Chemical and Biomolecular Engineering , University of Delaware , 150 Academy St. , Newark , DE , USA 19716 . Email: bxu@udel.edu

## Abstract

Due to the low volatility and highly oxygenated nature of biomass derived feedstocks, biomass upgrade reactions are frequently conducted in the presence of solvent to improve substrate mass transfer to the catalyst surface. However, relevant catalyst characterization techniques are most often performed in vacuum or inert gas environments, where the effect of solvent on the catalytic sites is ignored. Comparatively, characterization techniques in the presence of solvent are relatively rare, which poses challenges in developing structure–activity relationships for liquid phase reactions. In this perspective, commonly utilized techniques for probing the solid–liquid interface are briefly covered, with a focus on the role of solvent on zeolite and solid acid catalysis. New applications of techniques are proposed, most notably with ATR-FTIR, in the context of extracting thermodynamic information for the further understanding of the role of solvent on broadly applicable catalyst properties, such as acidity, and to develop structure–activity relationships for solid catalysts in solvent.

## Abstract 2

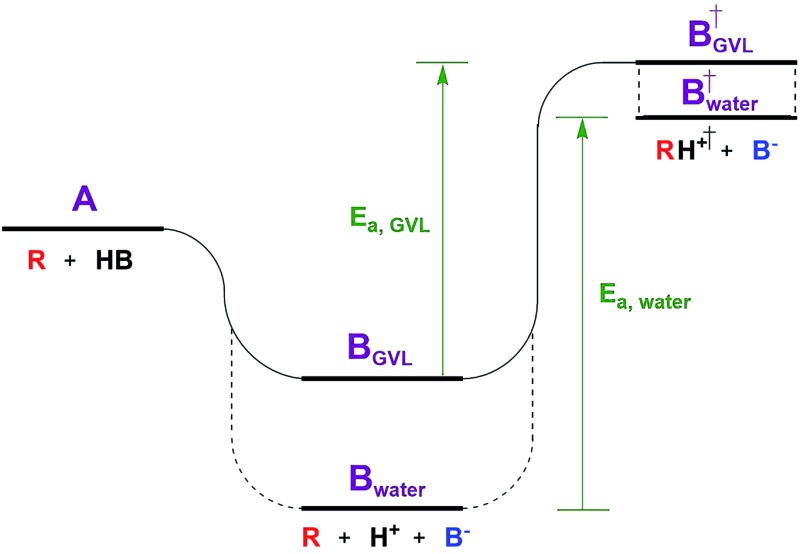
Due to the low volatility and highly oxygenated nature of biomass derived feedstocks, biomass upgrade reactions are frequently conducted in the presence of solvent to improve substrate mass transfer to the catalyst surface.

## 1. Introduction

Many biomass upgrade reactions are conducted in a solvent due to the highly oxygenated nature of the feedstock.^1^–^4^ This results in the heterogeneous catalytic active sites existing at a solid–liquid interface, where the solvent can modify surface and adsorbate energetics. Even when the solvent does not play a direct role in the reaction mechanism, it could stabilize or destabilize adsorbates, intermediates, and transition states, often leading to markedly different rates and selectivities.^2^,^5^–^9^ However, solvent effects are poorly understood because catalyst characterization techniques, such as probe molecule adsorption in FTIR, are most often conducted under vacuum or in the vapor phase.^10^,^11^ Understanding the role of solvent on catalytic sites is key to establishing liquid phase structure–activity relationships.

Currently, there is need for insight into fundamental liquid phase thermodynamic properties, *i.e.*, how the solvent choice affects the adsorption energies, solvation energies, and sorbate–sorbate interactions on the catalyst surface, in non-ideal environments like zeolite pores, and in bulk solution, as well as how these thermodynamic properties drive phase equilibria.^2^,^5^–^7^ In the liquid phase, a major challenge is to decouple these thermodynamic properties to understand how the solvent choice affects each of these terms individually. Thermodynamic insights pave the way to understanding the effect of solvents on the rate and product distribution, as activation barriers of individual elementary steps are closely correlated with the energetics of the reaction, *e.g.*, *via* the Brønsted–Evans–Polanyi (BEP) relation.^12^

## 2. Example reactions with significant solvent effects

**Fig. 1:**
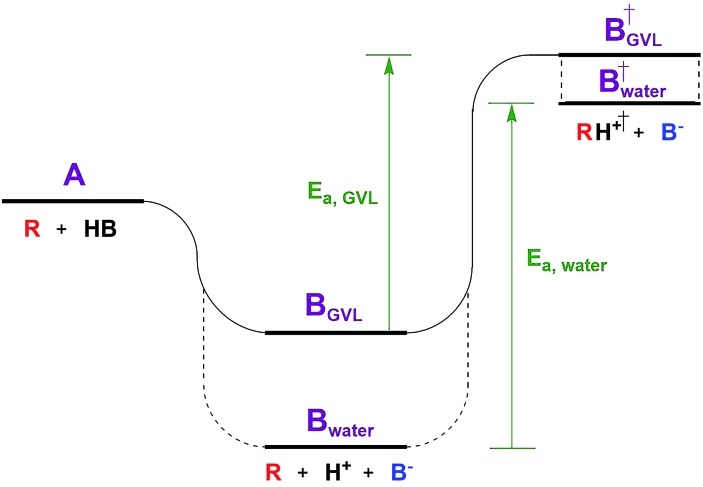
Reaction coordinate diagram depicting the effect of solvent.

The Dumesic group showed that turnover frequencies (TOFs) for the Brønsted acid catalyzed dehydration of xylose to furfural increased by 1–2 orders of magnitude in gamma valero lactone (GVL) compared to water over a wide range of homogeneous and solid acid catalysts, including zeolites.^5^ The disparity between solvents was less significant for weak acids.The low reactivity in water was attributed to water's greater ability to stabilize protons in solution compared to its ability to stabilize the transition state (TS) of the rate determining step (RDS, Fig. 1). The free energy of the dissociated acid (step B) and the free energy of the TS (step B^†^) can be significantly affected by the solvation of the surrounding liquid. GVL likely cannot stabilize protons as well as water, leading to a higher energy trough before the TS of the RDS. The relative amount that the solvent stabilizes the TS *versus* the dissociated acid affects the activation energy, and in turn the reaction rate. This work underlines the significant impact solvent can have on the stability of substrates, intermediates, transition states, and products, transforming the energy landscape, and leading to changed activation barriers.

Solvent effects are often discussed on two scales: one is microscopic and involves individual molecule interactions with solvents,^2^ while the more macroscopic/ensemble approach involves lumping these microscopic interactions into activity coefficients in rate expressions.^13^ The microscopic view highlights the extensive number of thermodynamic considerations involved in understanding the solid–liquid interface.^2^ In liquid phase reactions, for a substrate (A) to adsorb to the catalyst surface, or to an active site, it must first displace a previously adsorbed solvent molecule (B). This seemingly simple process contains eight thermodynamic terms, including: the adsorption energy of both A and B to the site in vacuum, the solvation energy of adsorbed A and B, the solvation energy of desorbed A and B, and sorbate–sorbate interactions between A, B, and their neighbors.^2^ These thermodynamic terms are of a molecular scale in nature, *i.e.*, dealing with single substrate molecules interacting with a single active site. However, the next example highlights that in the case of zeolite chemistry there are also more macroscopic thermodynamic considerations – those involving phase equilibria.

Solvents have also been shown to have a dramatic impact on Lewis acid catalyzed reactions such as the isomerization of glucose to fructose over NaX and NaY zeolites.^6^ Over NaX, the Scott group measured a 95% decrease in the TOF when using a 4 mol% GVL in water mixture compared to pure water. Surprisingly, the effect was non-monotonic, as the TOF started to recover as the GVL concentration increased further beyond 4 mol%. The sharp decrease of the TOF at a low GVL concentration was attributed to GVL uptake into the NaX pores, where it competitively adsorbed with glucose for the Lewis acid sites. As the GVL concentration increased, the bulk solution became increasingly hydrophobic, leading to increased water uptake into the pores. Diffusion measurements showed that glucose prefers solvation by water over GVL, and thus an increase in water uptake into the NaX pores led to a commensurate increase in glucose uptake. The authors noted, however, that the trends in glucose uptake alone were not sufficient to explain the changes in the TOF, and that the presence of GVL in the pores could also change the orientation and structure of nearby water molecules, which are known to interact with activated complexes in many carbohydrate isomerization reactions.^14^1
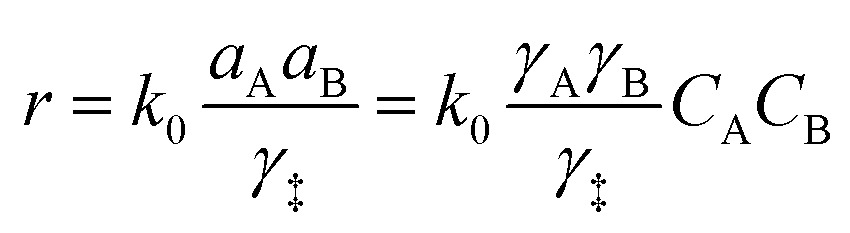



It is important to note that in non-ideal mixtures reaction rates are expressed in terms of activities, not in concentrations. Eqn (1) is the rate expression for a thermodynamically non-ideal reaction A + B → products, where *a*_i_ is the activity and *γ*_i_ is the activity coefficient of species i, the symbol ‡ represents the TS, and *k*_0_ is the thermodynamically ideal rate constant.^15^–^17^ Madon and Iglesia^13^ reasoned that a dependence on concentration in a non-ideal mixture only occurs when a kinetically relevant species (assuming it is A in this discussion) and the TS are similarly solvated, *i.e.*, when *γ*_A_ = *γ*_‡_, the rate scales with the concentration of A and the activity of B. The practical implications of the dependence on concentration or activity can be dramatic. For example, if the rate of a zeolite catalysed reaction is dependent on the concentration of A, then a solvent that increases the solubility (uptake) of A in the zeolite pores will increase the rate. However, the same does not necessarily hold for B, because an increase in the concentration of B often results in a corresponding decrease in the activity coefficient, leaving the activity (*γ*_B_*C*_B_) largely unaffected. This was demonstrated by Madon and Iglesia by equating the chemical potentials of liquid and vapor in a simple, two phase system.^13^ Assuming an ideal gas, this equality results in eqn (2), where *P*_g_ is the partial pressure of a component (assuming it is B) in the vapor phase, Δ*μ*° is the difference in the standard state chemical potentials between the gas and liquid phases (a constant), and *γ*_L_*C*_L_ is the activity of B in the liquid phase. If the pressure of B is set experimentally (hydrogen, for example), the left hand side of eqn (2) is constant, and an increase in the solubility (*C*_L_) in the solvent will be counteracted by a corresponding drop in the activity coefficient, *γ*_L_.^13^ This idea holds true for any two phases, including the bulk solvent and zeolite pores in a biomass reaction like the Scott example. However, for a non-ideal gas, or for kinetically relevant solution phase species, *e.g.*, sugars and furans, non-ideal terms (activity or fugacity coefficients) exist on both sides of the equality, and the relative solvent interactions in the two (or more) phases affect the activity, and thus the reaction rate. For an in-depth discussion of when activities or concentrations are kinetically relevant variables, the reader is directed to a paper by Madon and Iglesia.^13^2
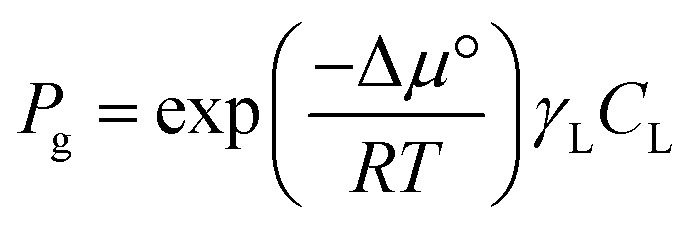



The examples from the Dumesic and Scott groups are a reminder that reaction rates in the liquid phase are driven, at least in part, by molecular scale thermodynamic considerations – terms that affect the activity coefficients, which are often lumped into the rate constant in experimental measurements, *i.e.*, adsorption energies, solvation energies, and sorbate–sorbate interactions, as well as by more macroscopic thermodynamics – phase equilibria that dictate the activity of the substrate in the phase containing the active site. The proper solvent choice also needs to promote a favorable substrate-active site interaction (which affects *γ*_‡_). In zeolites, much of the difficulty in understanding the solvent effects derives from van der Waals interactions with the pore walls. These interactions cause solvents to behave less “liquid-like”,^18^ and bulk solvent properties cease to apply.^19^ As seen in both examples, most efforts to understand the thermodynamics in this non-ideal environment have been on a case by case basis, to optimize a particular reaction product where solvents happened to play a critical role. Therefore, there is a need for liquid phase characterization techniques to generate a general understanding of the interactions among the solvent, the zeolite micropore environment, and the active sites.

## 3. State of the art in solid-liquid interface specific techniques

A variety of techniques are capable of probing the solid–gas interface, but solid–liquid interface characterization is experimentally challenging and many techniques are either not suitable, or are still in development for this application. Furthermore, the majority of techniques capable of probing the solid–liquid interface have focused on metal catalysts.^11^ Raman and UV Raman spectroscopy are frequently used to monitor metal precursor interactions with oxide supports,^20^ while surface enhanced Raman techniques like surface-enhanced Raman scattering (SERS)^21^ and shell-isolated nanoparticle-enhanced Raman spectroscopy (SHINERS) allow for traditional Raman spectra with enhanced surface selectivity due to metal particle plasmonic resonances.^22^,^23^ X-ray absorption spectroscopies (XAS), such as near edge (XANES) and fine structure (EXAFS), are typically used for vapor phase characterization, but they can be used to characterize the local structure and oxidation of metal catalysts in the presence of solvent.^11^,^24^–^27^ X-ray emission spectroscopy (XES) can provide information on the electronic structure, charge/spin densities, and the nature of ligands, and is more adept than XAS techniques at metal catalyst characterization in complex, *in situ* environments that do not require ultra-high vacuum (UHV).^11^,^28^,^29^ X-ray photoelectron spectroscopy (XPS) experiments have been conducted in the presence of water to study metal ions in solution and colloidal metal nanoparticles (NPs),^30^–^32^ although XPS has rarely been used for the *in situ* monitoring of the reaction progress in the liquid phase. UV-vis spectroscopy is commonly used for monitoring homogeneous metal complexes in solution, and characterizing metal oxides in the vapor phase, but is rarely applied to heterogeneous catalysts in the presence of a solvent.^11^ Electron microscopy (EM) techniques have been used to study metal NP growth in the presence of solvent, but have not been used to monitor catalysts during *in situ* reaction conditions.^33^ Scanning probe microscopies like scanning tunnelling microscopy (STM) and atomic force microscopy (AFM) are promising for solid–liquid interface imaging, and do not suffer from charging problems/surface alterations that can be caused by EM techniques, but are rarely integrated with other optical techniques like FTIR, especially for liquid phase systems.^11^ A recent review by Shi *et al.* covers the current state of these techniques.^11^

For characterizing the solid–liquid interface of zeolites and solid acid catalysts, solid-state NMR and attenuated total reflection (ATR)-FTIR are among the most frequently used and informative experimental techniques. Recently, customized NMR rotors have been designed to withstand conditions as extreme as 523 K and 200 bar, making solid state NMR a robust technique for the *in situ* monitoring of reactions.^34^,^35^ Additionally, solid state NMR with magic-angle spinning (MAS) is particularly well suited for differentiating substrates that are adsorbed *versus* those remaining in bulk solution.^6^ This is because adsorbed species typically result in significant peak broadening due to the restricted mobility or heterogeneities in the adsorbed structures.^36^ Unfortunately, the adsorbate peaks often do not shift significantly from those of their bulk counterparts, requiring peak deconvolution. Furthermore, the solvent can contribute to the NMR signal, and thus further deconvolution may be necessary. Selective detection of adsorbed species at the solid–liquid interface, in the presence of often overwhelming bulk species and solvents, is a general challenge for most spectroscopic methods, *e.g.*, NMR and ATR-FTIR. If quantitative information is desired, NMR has typically been preferred over ATR-FTIR, although a method for determining the liquid phase extinction coefficients of adsorbed pyridine on zeolites was developed in our recent work to circumvent this challenge.^37^ The method involved combining a batch experiment to measure the amount of pyridine adsorbed on a zeolite in a particular solvent, with a continuous flow experiment with a specific pyridine feed concentration to achieve the same equilibrium state as that of the batch experiment. The resulting extinction coefficients were dependent on the solvent choice, but universal for all zeolite framework structures. This was the first effort to develop ATR-FTIR into a quantitative technique for adsorbates, as typically normalized intensities are used to make semi-quantitative comparisons. Compared to solid state NMR, quantitative ATR-FTIR offers a simple experimental method to compare the concentrations of bulk and surface species and the uptake into zeolite pores. These concentrations can be compared to catalytic activity data to test the non-ideality of the system, and whether substrates and activated complexes have similar interactions (eqn (1)).

**Fig. 2:**
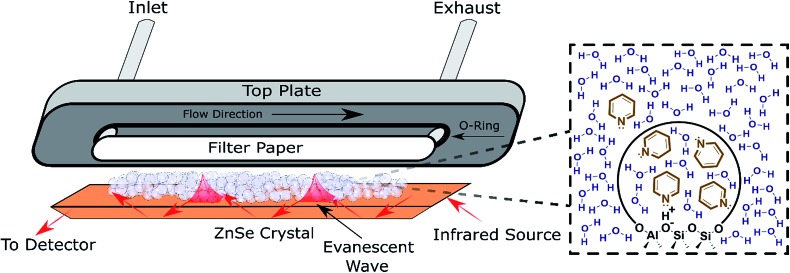
Schematic of an ATR flow cell. Inset: Depiction of the pyridine partition (concentration change) between the bulk solvent and the pores detected *via* ATR-FTIR.

ATR-FTIR spectroscopy has been employed to monitor reaction progress and identify adsorbates at the solid–liquid interface.^38^,^39^ To maximize the signal resulting from the solid–liquid interface, the penetration depth of the evanescent wave (typically 1–5 μm) should be saturated with the catalyst.^39^ Thus, the typical surface sensitive ATR-FTIR design is a flow cell with the catalyst particles deposited directly on the ATR crystal (Fig. 2). The addition of a layer of inert filter paper can be used to prevent the catalyst from flowing out of the exhaust with the solvent, and avoid the difficulty involved in making stable films.^40^ A thorough review covered pertinent ATR cell design principles.^38^ While the design in Fig. 2 has been used to monitor reactions and identify adsorbates,^41^ characterization with ATR-FTIR has most frequently been used for supported transition metal catalysts, with Pt/Al_2_O_3_ and Pd/Al_2_O_3_ as the most extensively characterized heterogeneous catalysts in the liquid phase in the literature.^42^–^47^ Furthermore, characterization *via* ATR-FTIR is most commonly employed to optimize a particular reaction, instead of building an understanding of general catalyst properties in the presence of solvent. Acidity is a property applicable to a broad spectrum of reactions that has not been thoroughly characterized in the liquid phase, which will be employed as a representative example in the next section. However, most of the experimental strategies are applicable to a variety of catalysts, *e.g.*, supported metal catalysts.

## 4. Perspectives

Developing structure–activity relationships for solid catalyst mediated liquid phase reactions is dependent on decoupling the thermodynamic terms that arise in the presence of a solvent. There are three molecular scale terms directly involving the active site: solvation, adsorption, and sorbate–sorbate interactions, as well as macroscopic phase equilibria considerations. These thermodynamic factors determine the equilibrium concentrations and activities of the substrate or probe molecule in all phases. Experimentally, the concentrations of the bulk and adsorbed probe molecules or substrates can be estimated using ATR-FTIR with known extinction coefficients or using solid state NMR in the presence of a solvent.

**Fig. 3:**
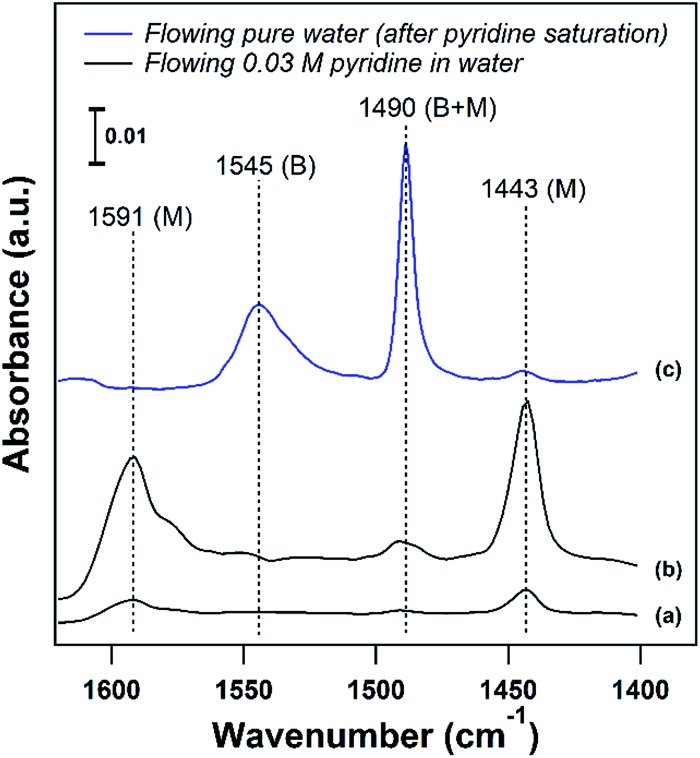
(a) ATR-FTIR spectra of flowing 0.03 M pyridine in water through the ATR cell devoid of catalyst at 25 °C and (b) through a bed of hydrophilic Si/ZSM-5 at 25 °C. (c) Pyridine adsorbed on H/ZSM-5 (Si/Al = 11) after purging with pure water at 75 °C. (B) Brønsted and (M) molecularly adsorbed/bulk.

An ideal starting point is to decouple the effect of the solvent on the activities of the reactants in the vicinity of the active sites from the effects on the properties of an individual substrate – active site interaction (*γ*_‡_). As discussed in the example from the Scott group,^6^ this is particularly relevant in porous catalysts like zeolites where the solvent composition can significantly affect the concentration of the substrate in the pores (the partition coefficient), in the vicinity of the acid sites during the reaction. Quantifying the partition of substrates in the bulk *vs.* near active sites is key to obtaining the intrinsic activity (or TOF) of the catalytic sites. To this end, ATR-IR could be an effective tool to differentiate substrates in microporous materials from those in the bulk, as illustrated by the enhanced signal of the vibrational bands corresponding to pyridine in the presence of a non-acidic siliceous ZSM-5 zeolite in our preliminary study (Fig. 3a and b). These spectra depict a dilute pyridine in water solution flowing through the cell illustrated in Fig. 2 devoid of catalyst (Fig. 3a) and with 25 mg of hydrophilic siliceous ZSM-5 (Fig. 3b). The area of the 1444 cm^–1^ peak, corresponding to pyridine that is either in the bulk phase or molecularly adsorbed to the pore walls, is approximately one order of magnitude greater in the zeolite than in the bulk. Thus, the partition in water results in roughly a tenfold increase in the pyridine concentration in the vicinity of acid sites (Fig. 2, inset).

The strength of the acid sites in different solvents could be characterized with temperature programmed desorption (TPD) experiments with a base probe molecule like pyridine in the presence of solvent. For acidic catalysts, liquid phase pyridine TPD can provide insight into the density and strength of Brønsted acid sites (BASs) in the presence of a solvent. In agreement with the corresponding vapor phase TPD, pyridine is more weakly bound to Lewis or molecularly adsorbed sites than to BASs, and can be removed at temperatures below 100 °C on zeolites in the liquid phase. This is demonstrated in Fig. 3c, where adsorbed pyridine on H/ZSM-5 (Si/Al = 11) was purged with pure water to attempt to remove pyridine from BASs (1547 cm^–1^) and molecularly adsorbed sites (1444 cm^–1^) at 75 °C. The majority of pyridine was removed from molecularly adsorbed sites below the solvent boiling point, while pyridine adsorbed on BASs was unaffected. This allows for a clear separation, where desorption of pyridine above the solvent boiling point can be assigned to BASs, and liquid phase TPD can characterize the BAS strength in the presence of solvent.

**Fig. 4:**
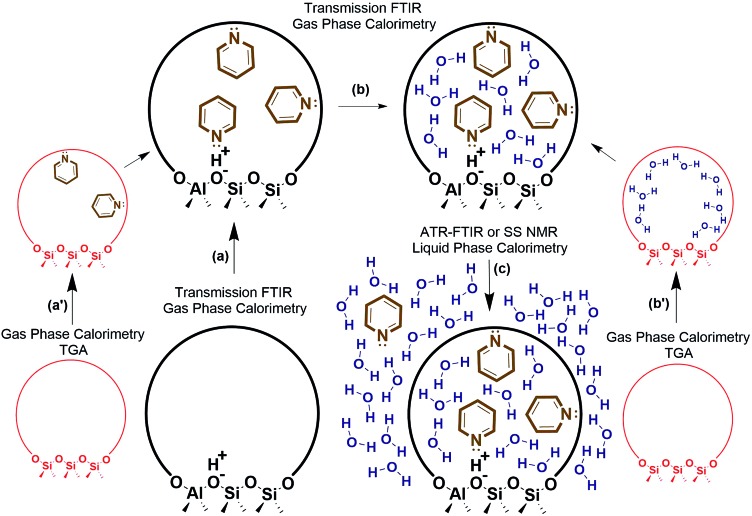
Experimental outline for estimating liquid phase adsorption, solvation, and interaction energies.

Decoupling the thermodynamics of adsorption, solvation, and sorbate interactions surrounding the active site could be facilitated through a combination of calorimetry, thermogravimetric analysis (TGA), transmission FTIR, ATR-FTIR, solid state NMR, and density functional theory (DFT) calculations. A general outline is provided in Fig. 4, where vapor phase calorimetry measurements in steps a, a′, b and b′ can provide the heats of adsorption of the probe molecules, substrates, or solvent molecules to the active sites. For catalysts containing multiple types of adsorption site, the calorimetric data would require deconvolution using spectroscopic techniques such as quantitative transmission FTIR, ATR-FTIR, or solid state NMR (steps a–c), and simple control experiments such as calorimetry on the support material, on purely siliceous zeolites (steps a′ and b′), zeolites with varying Si/Al, or metal catalysts with varying loading. Solvation energies could be approximated *via* liquid phase calorimetry paired with ATR-FTIR with known extinction coefficients or with solid state NMR to quantify the amount of a probe molecule or substrate in all phases (step c). In liquid phase systems, it is particularly important to ensure the multiple phases are at equilibrium, and ATR-FTIR with continuous scanning can help estimate the time required for achieving the final state. It is worth noting that the proposed experimental outline consisting of calorimetry, transmission FTIR, ATR-FTIR, probe molecule TPD, and NMR can be applied to a variety of solid catalysts to elucidate the role of solvent at the solid–liquid interface. Eventually, these solvation and adsorption energies could be compared to those of computational modelling estimates. Accurate computational models would reduce or eliminate the need for this laborious experimental process, and could efficiently screen solvent thermodynamic properties.

A more ambitious goal is to understand and develop accurate computational models capable of predicting the effect of solvents on the rate and product distribution of liquid phase reactions. This requires knowledge of the interaction between the solvent molecules and the activated complex (within the framework of the transition state theory), which strictly speaking cannot be obtained based purely on thermodynamic properties. In the absence of any reliable first principles based models, the semi-empirical correlation between the reaction energy and the activation barrier, or the BEP relation,^12^ could be considered as a first approximation. Experimentally determined activation barriers in various solvents could yield the raw data to extract quantitative, albeit empirical, BEP relations, which will also pave the path to gain a more in depth understanding of the interplay between solvents and activated complexes.

Improved understanding of the solid–liquid interface in thermo-catalytic processes could also benefit related fields such as electro-catalysis. Due to an applied potential, and a distinct phase of solvent near the electrode surface (the electrochemical double layer), the concentrations of species at the electrochemical interface could differ substantially from those in the bulk, and the interface concentrations are key to determining the intrinsic rate and selectivity of the reaction. As a result, interfacial specific spectroscopic techniques, such as surface enhanced infrared absorption reflection spectroscopy, have become indispensable tools in understanding electrode surface mediated reaction mechanisms and the impact of electrolytes.^48^–^52^

## Conflicts of interest

There are no conflicts to declare.
